# Numerical data on fire in the cavity of naturally ventilated double skin façade with Venetian blinds

**DOI:** 10.1016/j.dib.2022.108859

**Published:** 2022-12-28

**Authors:** Youxian Huang, Siegfried Yeboah, Jingjing Shao

**Affiliations:** a Department of Architecture and Built Environment, Faculty of Science and Engineering, The University of Nottingham Ningbo China, 199 Taikang East Road, Ningbo 315100, PR China; b School of the Built Environment and Architecture, London South Bank University, 103 Borough Road, London SE1 0A, UK; c Ningbo University of Technology, 201Fenghua Road, Ningbo, Zhejiang 315211, PR China

**Keywords:** Building fire safety, Double skin façade, Fire Dynamics Simulation, Venetian blinds

## Abstract

This Data Article presents simulation data and methodology on fire in the cavity of naturally ventilated Double Skin Façade (DSF) with Venetian blinds. The simulation data includes glazing surface temperature data and the Input and Output Source Code files. The data for the validation of the model is also presented along with its methodology, input source code file and output temperature results. The comprehensive methodology used to obtain this data from the National Institute of Standards and Technology's (NIST) Fire Dynamics Simulator (FDS) and PyroSim are presented. The data presented can provide theoretical benchmarks for architects, engineers, researchers, and designers when incorporating Venetian blinds in DSFs. It can also help fire fighters and engineers to theoretically assess the spread of fire in buildings with DSFs incorporating Venetian blinds.

**Specifications Table**

**Table:** 

Subject	Engineering
Specific subject area	Fire Safety Engineering
Type of data	Table Image Chart Graph Figure Input and Output Simulation Data Files
How the data were acquired	Data was acquired from the simulation of a 5-storey building with multi-storey type DSF incorporating Venetian blinds. The model was created and simulated in PyroSim Version 2021.1.0224 and Fire Dynamics Simulator version 6.7.5. Fuel for the fire was Polyurethane GM27. Heat Release Rate was 5MW. Thermocouples on the inner and outer glazing of each floor measured DSF cavity temperatures. Visualization of the simulation was achieved using Smokeview. The computer used for the simulation was ASUS U4100 laptop with Windows 10 Operating System, 4G memory and Intel ® Core™ i5 - 7200U CPU @ 2.50GHz processor.
Data format	Raw
Description of data collection	The glazing surface temperatures were obtained from thermocouples installed on each floor. On each floor, three thermocouples were installed on the inner glazing and three on the outer glazing surfaces in the cavity of the DSF. The Input and Output Source Code Files showing the simulation process is also included.
Data source location	Institution: The University of Nottingham Ningbo China City/Town/Region: Ningbo, Zhejiang Province Country: P. R. China
Data accessibility	Repository name: Digital Commons data (Mendeley Data) Data identification number: doi: 10.17632/cdc5wfmhs2.2 Direct URL to data: https://unnc.digitalcommonsdata.com/datasets/cdc5wfmhs2
Related research article	Huang Y, Yeboah S, Shao J. Numerical investigation of fire in the cavity of naturally ventilated double skin façade with Venetian blinds. Building Services Engineering Research and Technology. 2022;0(0). doi:10.1177/01436244221129763 https://journals.sagepub.com/doi/10.1177/01436244221129763

## Value of the Data

• This dataset provides glazing surface temperature data, Input and Output Source Code files on fire in the cavity of naturally ventilated Double Skin Façade (DSF) with Venetian blinds and can provide theoretical benchmarks for architects, façade engineers, built environment researchers, and building designers when incorporating Venetian blinds in DSFs.
• The dataset including the detailed methodology on the modelling and simulation of fire in sustainable buildings incorporating DSF technology will be useful to researchers, engineers, and designers involved with façade engineering and related fire safety.
• Since fire events are of high consequence and uncertainty and present challenges with validation of fire models, this dataset and associated methodology can provide avenues for fire engineers to validate their theoretical models and also serve as a benchmark for experimental fire models.

## Objective

This dataset backs the results presented in Huang et al. [1]. The purpose is to provide transparency to the scientific work done by the authors and to provide opportunities for building services and fire engineering researchers to replicate the work done and to validate their own models. Along with the detailed methodology and the source codes provided, the purpose of this dataset is to allow reproducibility and reuse in furtherance of future related studies in fire protection for green and sustainable buildings. Since the original article is to serve us a benchmark for other theoretical studies beneficial to fire fighters and engineers, access to the dataset behind this benchmark will be invaluable. Members of professional institutions such as the Chartered Institution of Building Services Engineers (CIBSE), the Society of Façade Engineering and the Institution of Fire Engineers (IFE) will also benefit from the scientific and engineering numerical methods used in obtaining the fire performance data of this DSF building.

## 3 Data Description

### 3.1 Description of the Glazing Surface Temperature Data

The temperature data are glazing surface temperatures in °C obtained from Thermocouples installed on the inner and outer glazing surfaces. Three Thermocouples were installed vertically, 0.1 m apart on the inner glazing and another three were installed on the outer glazing of the DSF on each floor. The data was collected for various scenarios depicting the Venetian blind position and slat angle opening. The Venetian blinds were positioned in three different ways, 0.5 m away from the inner glazing, middle and 0.5 m away from the outer glazing of the DSF cavity. The slat angles for the Venetian blinds were opened at 135°, 90°, 45° and 0°, respectively for the varied Venetian blind positions. The simulation time for each scenario was 100 s. The surface temperature data are presented in the Mendeley Data repository [2] on a .xlsx file captioned ‘Temperature Data-Fire in DSF with Venetian Blinds’. In this file, three tabs depicting data from thermocouples installed on the inner and outer glazing surfaces for floors 2, 3 and 4, respectively are provided. On floor 2, the thermocouples used to obtain the inner glazing the surface temperatures were TC 14, TC 15, and TC 16, respectively. For the outer glazing surface temperature, the thermocouples were TC 04, TC 05, and TC 06, respectively. For floor 3, the inner glazing thermocouples were, respectively, TC 17, TC 18, and TC 19 and the outer glazing thermocouples were TC 07, TC 08, and TC 09, respectively. For floor 4, the inner glazing thermocouples were TC 20, TC 21, and TC 22, respectively and the outer glazing was TC 10, TC 11, and TC 12, respectively. It is important to note that the 5-storey building model was sectioned from bottom up as ground floor, floor 1 (fire room), floor 2, floor 3 and floor 4, respectively. On the tab labelled *‘Temperature on the Floor 2’*, there are 14 sets of data obtained over the simulation time of 100 s. In the first column of each dataset is the entry for the simulation time from 0 s to 100 s with an interval of 0.1 s and covering 1000 dataset points. Columns B to G shows glazing surface temperature data for 1 MW HRR fire in the DSF cavity when there was no Venetian blind installed. A similar simulation was carried out with a 5 MW HRR fire when there was no Venetian blind installed and the surface temperature data obtained are shown in columns I to N. When the Venetian blind was inserted in the cavity of the DSF, only a 5 MW HRR fire was used in the simulation. Columns O to AI, shows the glazing surface temperatures when the Venetian blind was opened at an angle of 135°. At this slat opening angle, the Venetian blind was, respectively positioned 0.5 m away from inner glazing, in the middle of the DSF cavity, and 0.5 m away from outer glazing. For the same positions with respect to the Venetian blind in the cavity of the DSF, glazing surface temperatures for when the blind opening angle was 90°, 45° and 0° are, respectively shown in columns AJ to BD, BE to BY and BZ to CT.

The same simulation time entry is for tabs labelled *‘Temperature on the Floor 3’* and *‘Temperature on the Floor 4’*, respectively all in the .xlsx file captioned ‘‘Temperature Data-Fire in DSF with Venetian Blinds’’ in the Mendeley Data repository [2]. On both tabs, similar entries as presented for the tab labelled *‘Temperature on the Floor 2’*, is presented. The only difference is the thermocouple numbers as outlined above. Also, in the spreadsheets for each tab, there are colour codes to distinguish between the glazing surface temperatures for the simulation with no Venetian blinds, those with Venetian blind position and their related slat opening angles. For the results with no Venetian blinds, the HRR for the fires (1 MW and 5 MW, respectively) used in the simulation are shown. For those with Venetian blind, 5MW HRR fire was used, and it is not indicated. Graphical plots of the results are presented in Huang et al. [1].

**Fig. 1:**
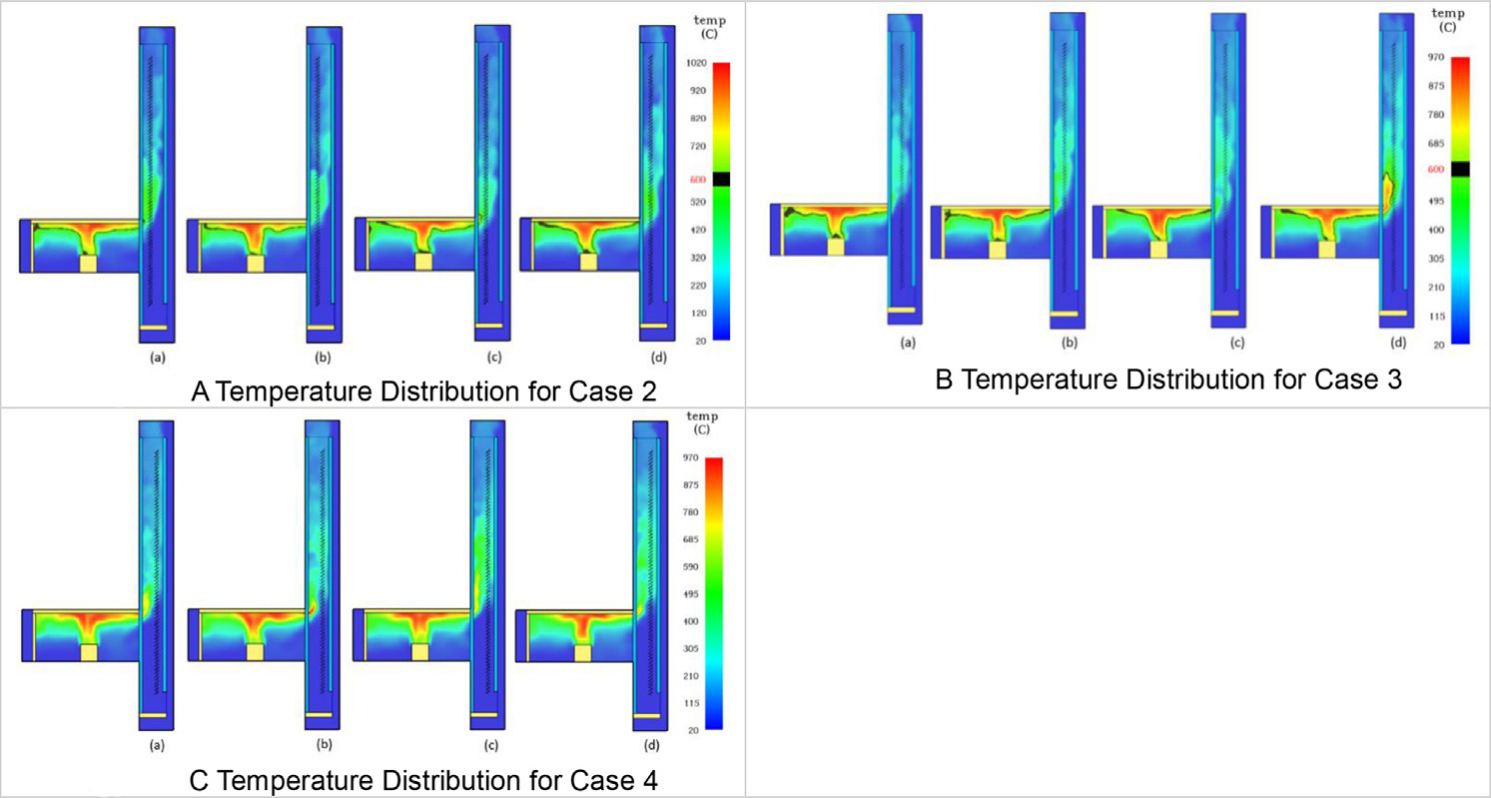
A Temperature Distribution for Case 2. Fig. 1B Temperature Distribution for Case 3. Fig. 1C Temperature Distribution for Case 4.

**Fig. 2:**
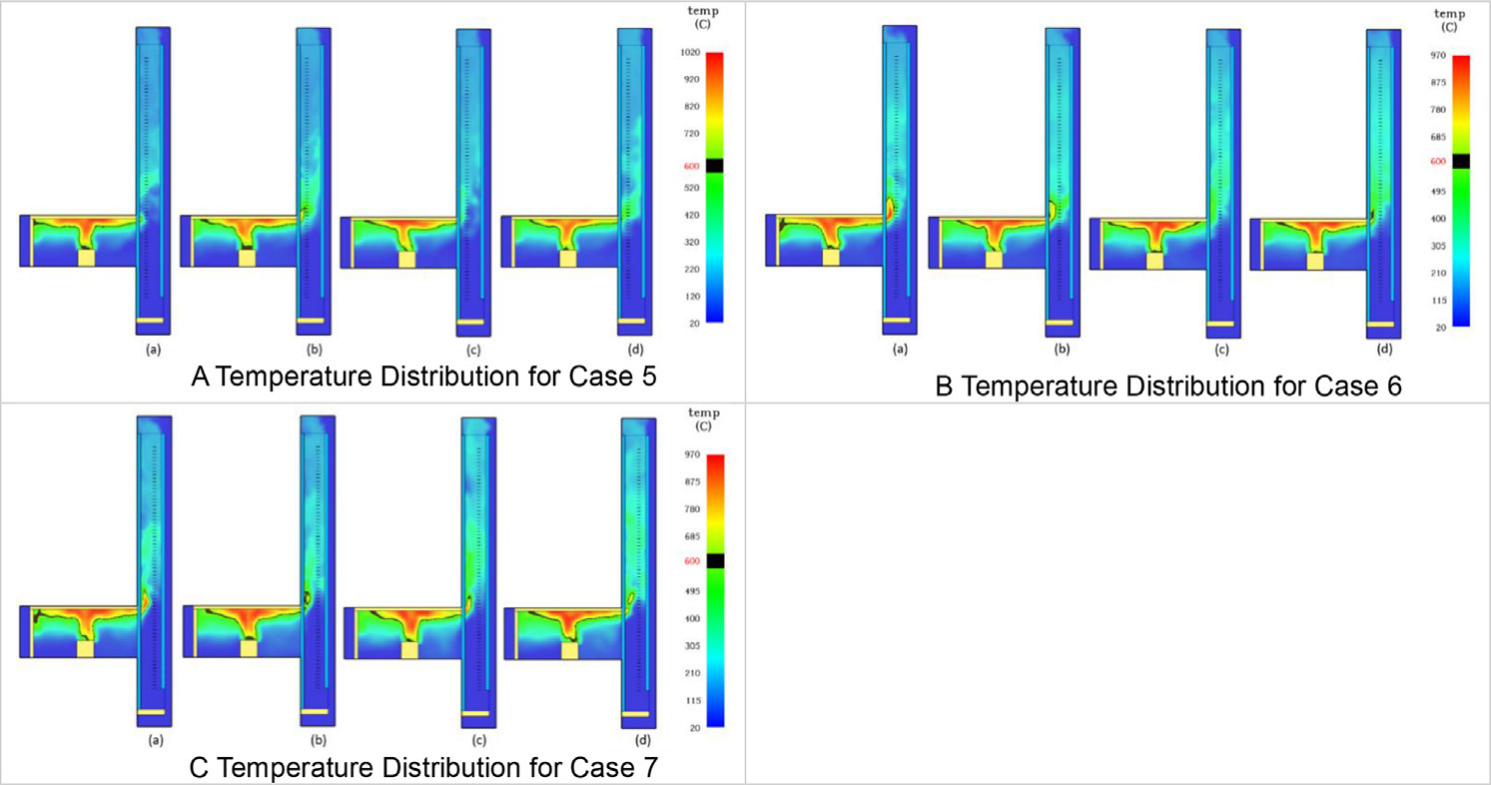
A Temperature Distribution for Case 5. Fig. 2B Temperature Distribution for Case 6. Fig. 2C Temperature Distribution for Case 7.

**Fig. 3:**
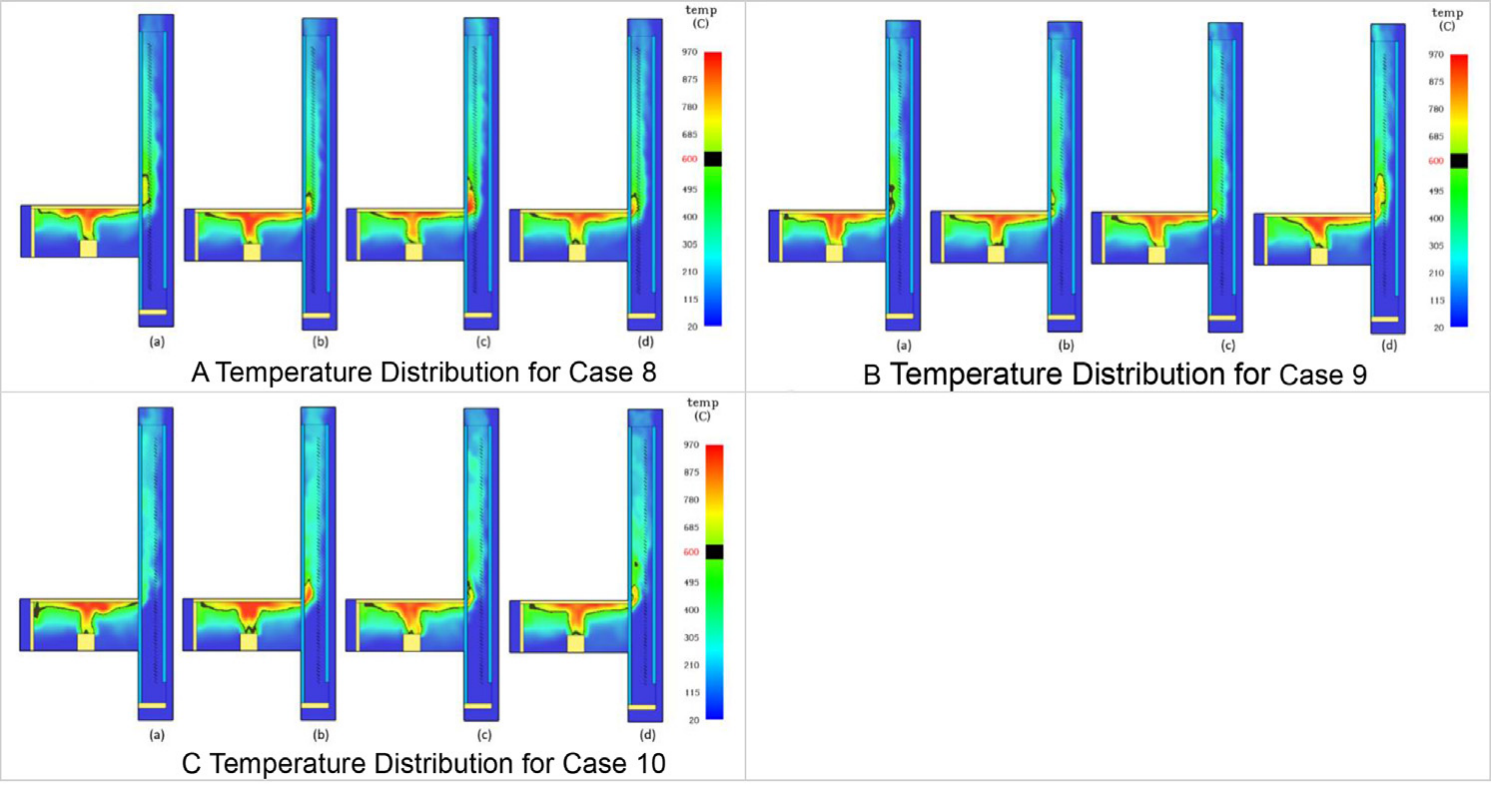
A Temperature Distribution for Case 8. Fig. 3B Temperature Distribution for Case 9. Fig. 3C Temperature Distribution for Case 10.

**Fig. 4:**
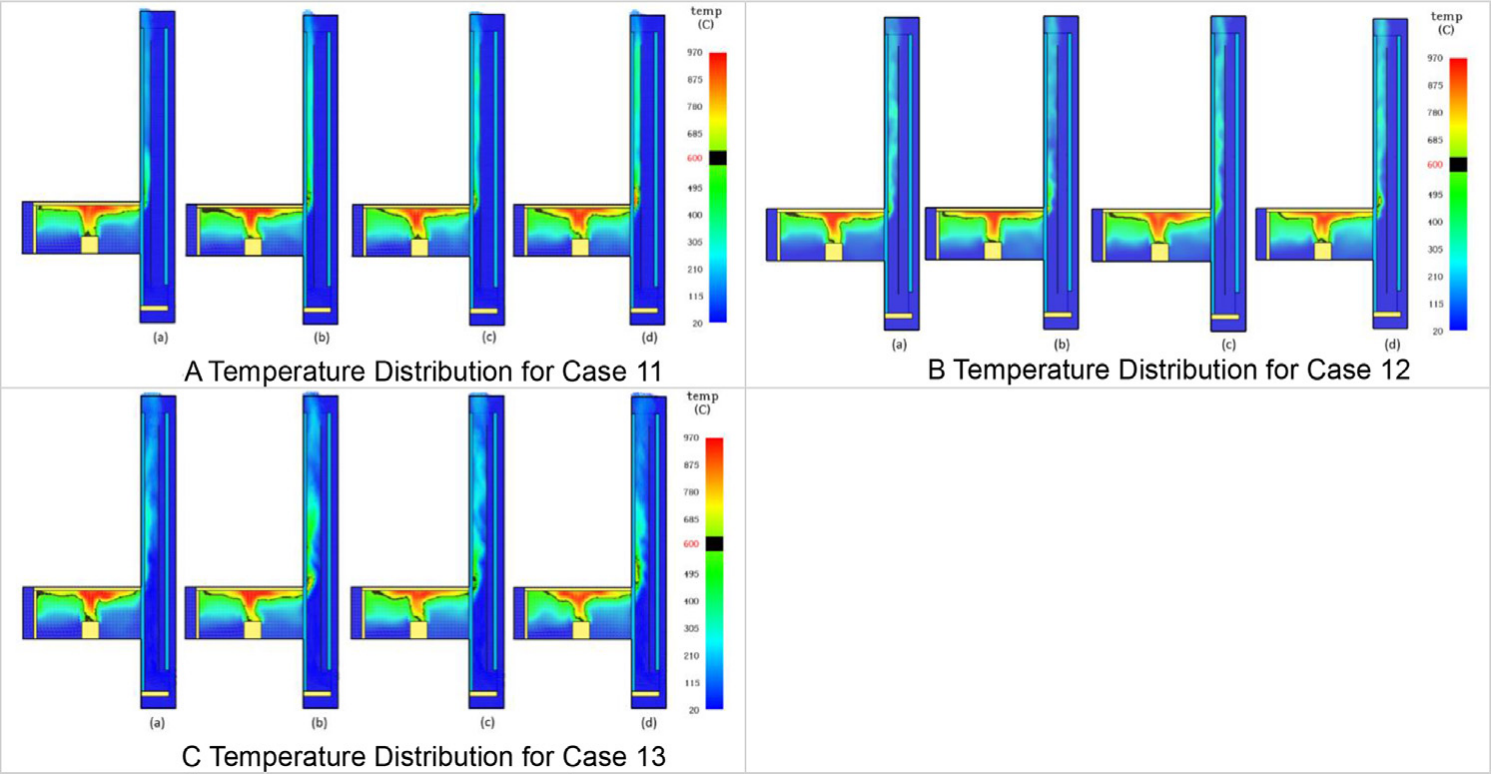
A Temperature Distribution for Case 11. Fig. 4B Temperature Distribution for Case 12. Fig. 4C Temperature Distribution for Case 13.

**Fig. 5:**
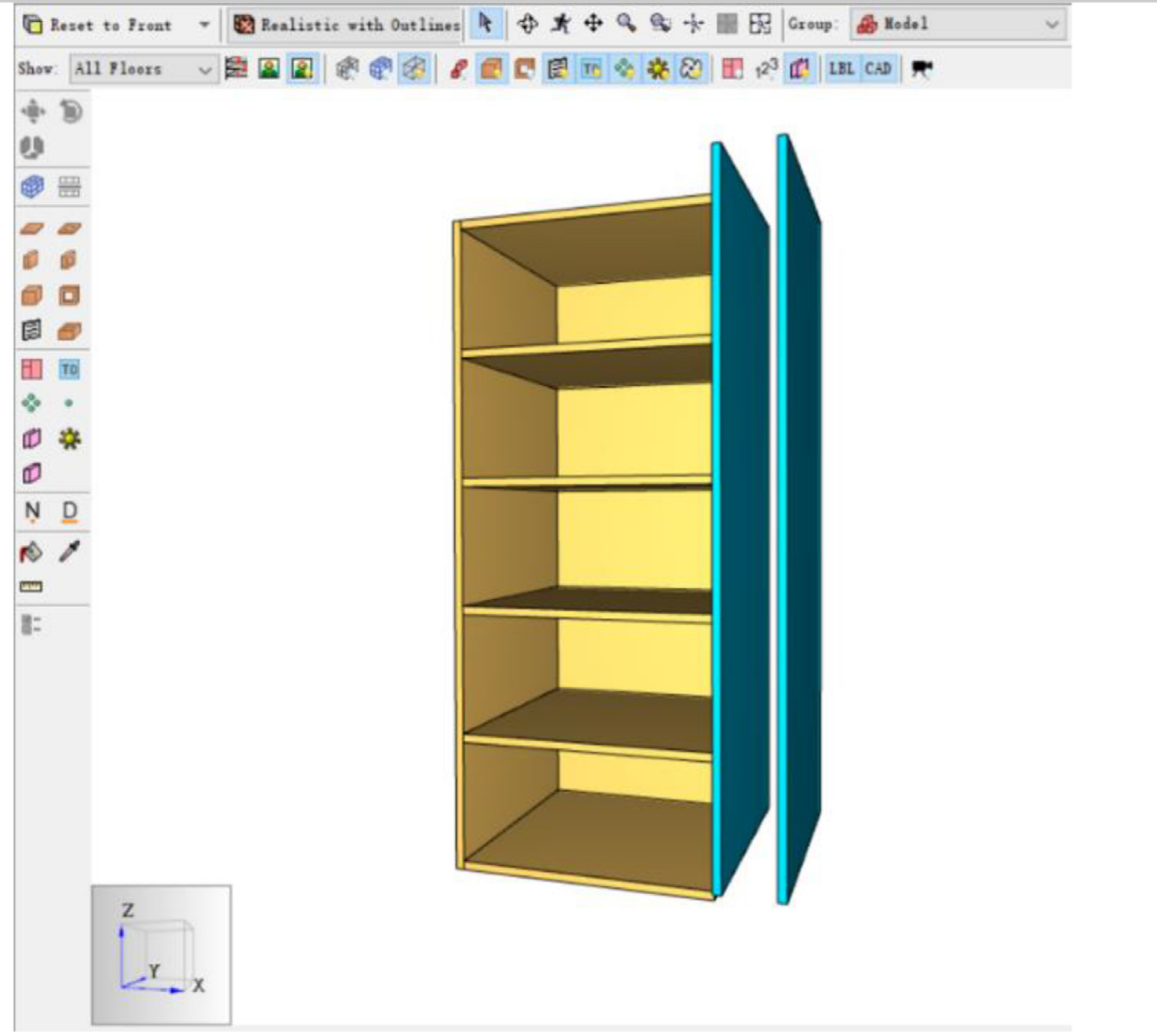
Building model with Inner and Outer DSF Glazing Layers.

**Fig. 6:**
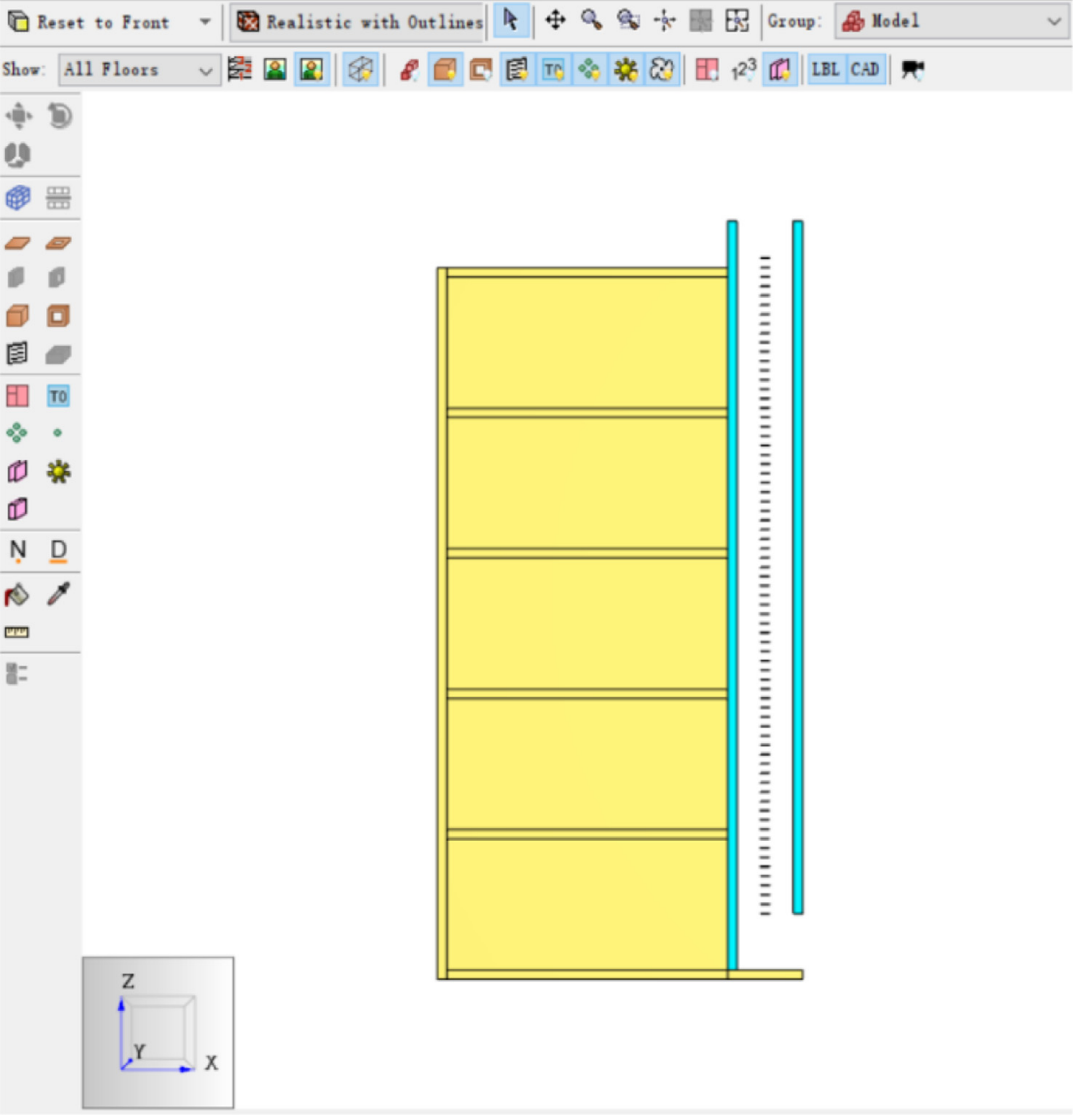
Venetian Blinds installed in the DSF Cavity.

**Fig. 7:**
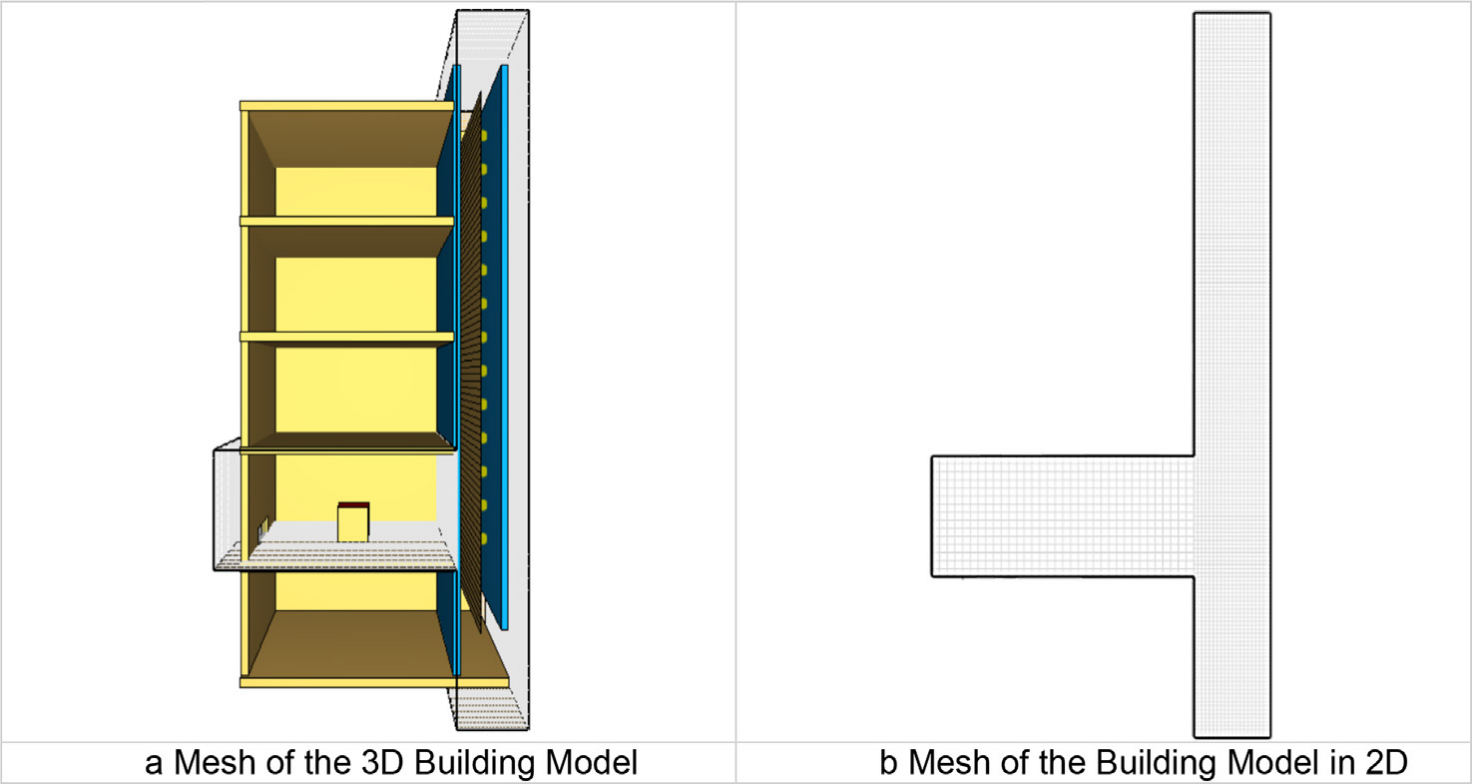
a Mesh of the 3D Building Model. Fig. 7b Mesh of the Building Model in 2D.

**Fig. 8:**
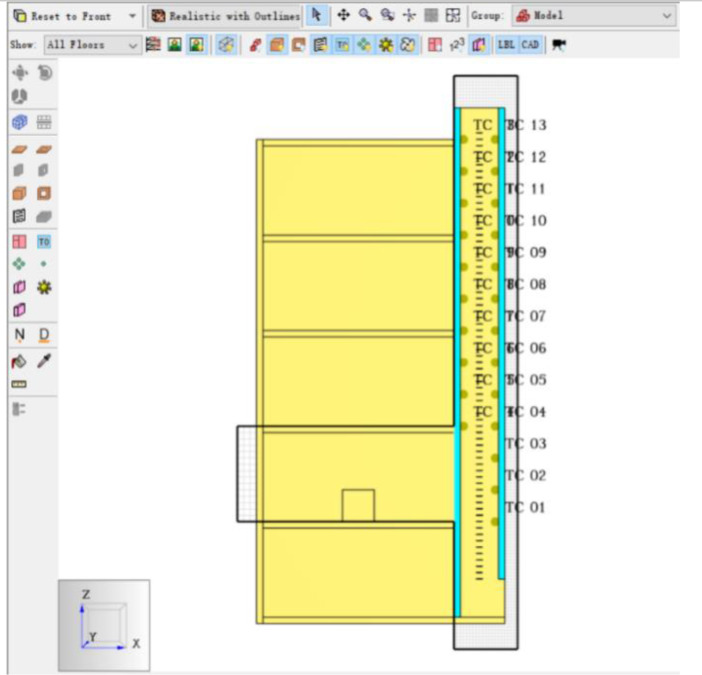
Thermocouples Installed in DSF Cavity.

**Fig. 9:**
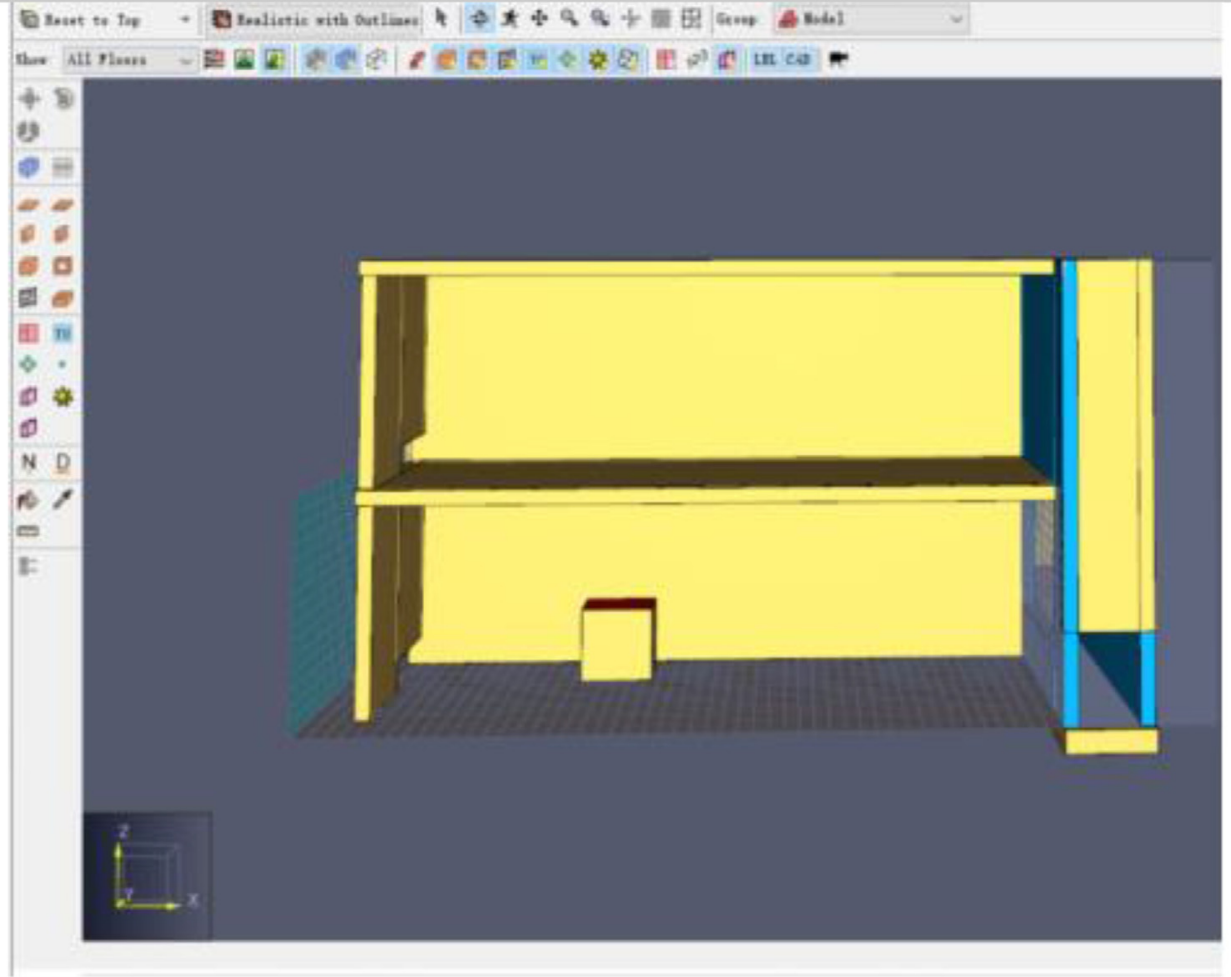
Model with Inner and Outer DSF Glazing.

**Fig. 10:**
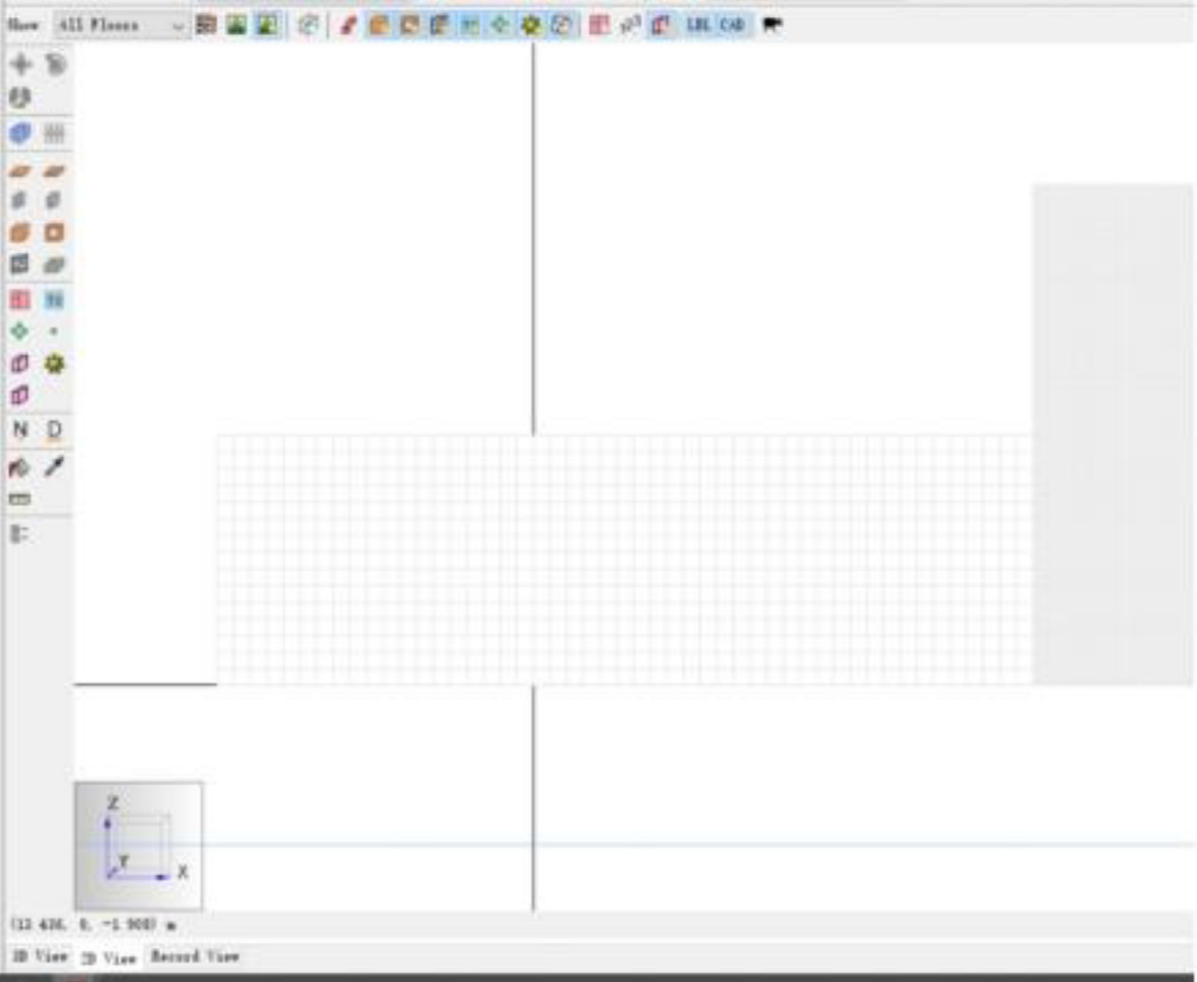
Mesh.

**Fig. 11:**
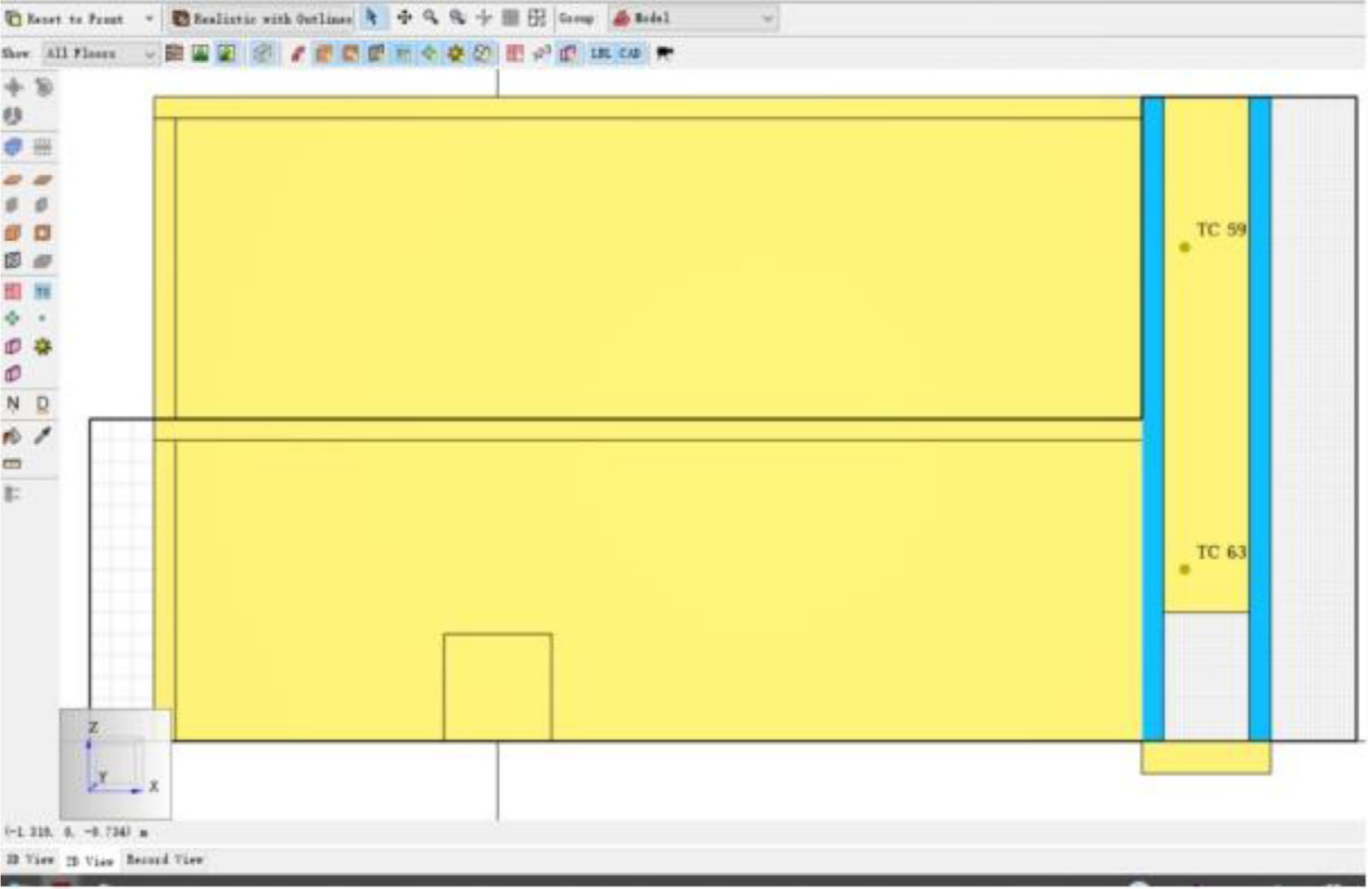
Installed Thermocouples.

The Table and Figures in this data article, along with their descriptions are as follows: Table 1 - Key to Figs. 1(A–C) to 4(A–C); Fig. 1A - Temperature Distribution for Case 2; Fig. 1B - Temperature Distribution for Case 3; Fig. 1C - Temperature Distribution for Case 4; Fig. 2A - Temperature Distribution for Case 5; Fig. 2B -Temperature Distribution for Case 6; Fig. 2C - Temperature Distribution for Case 7; Fig. 3A - Temperature Distribution for Case 8; Fig. 3B - Temperature Distribution for Case 9; Fig. 3C - Temperature Distribution for Case 10; Fig. 4A - Temperature Distribution for Case 11; Fig. 4B - Temperature Distribution for Case 12; Fig. 4C - Temperature Distribution for Case 13; Fig. 5 - Building model with Inner and Outer DSF Glazing Layers; Fig. 6 - Venetian Blinds installed in the DSF Cavity; Fig. 7a - Mesh of the 3D Building Model; Fig. 7b - Mesh of the Building Model in 2D; Fig. 8 - Thermocouples Installed in DSF Cavity; Fig. 9 - Model with Inner and Outer DSF Glazing; Fig. 10 – Mesh; Fig. 11 - Installed Thermocouples.

**Table 1:** Key to Figs. 1(A–C) to 4(A–C).

Simulation Cases	Venetian Blind Position in DSF			Venetian Blind Opening Angle			
	Close to the Inner Glazing	Middle	Close to the Outer Glazing	135°	90°	45°	0°
Case 2	**√**			**√**			
Case 3		**√**		**√**			
Case 4			**√**	**√**			
Case 5	√				**√**		
Case 6		**√**			**√**		
Case 7			**√**		**√**		
Case 8	√					**√**	
Case 9		**√**				**√**	
Case 10			**√**			**√**	
Case 11	√						**√**
Case 12		**√**					**√**
Case 13			**√**				**√**

### 3.2 Description of the Input and Output Source Code Files

In the folder captioned Fire in the DSF with Venetian Blind - Input Data in the Mendeley Data repository [2] are FDS input files with the file extension “. fds”. In this folder the “. fds” codes for the various slat angle openings and corresponding Venetian blind positions along with that for the sensitivity analysis and DSF without Venetian blinds are provided. These codes are openable in Microsoft Notepad editor.

The output data files are found in the folder captioned Fire in the DSF with Venetian Blind - Output Data in the Mendeley Data repository [2]. The files in this folder are “. smv” and “_devc.csv” files. Based on the blind slat opening angles and blind positions, the input data generated the corresponding output results. The “. smv” file allows visualization of the simulation results in the Smokeview and can be opened in Microsoft Notepad editor. While the “_devc.csv” file contains the numerical values for the calculation of the & DEVC line in the FDS. The “_devc.csv” file are individual comma-separated values files of the glazing surface temperatures obtained for each thermocouple under each simulation case.

### 3.3 Simulation Results – Temperature Distribution

2D slices from the Output menu selected from Animated Planar Slices were used to the visualize the results. Results from this approach for the temperature distribution in the DSF cavity are shown in Figs. 1(A–C) to 4(A–C). In Fig. 1(A–C), the Ventetian Blind was opened at 135° and placed in the DSF cavity, 0.5 m away from inner glazing, Middle, and 0.5 m away from outer glazing, respectively. In Figs. 2(A–C), 3(A–C), and 4(A–C), the Venetian blind was positioned as in Fig. 1(A–C), however the slat opening angles were 90°, 45° and 0° are, respectively. So in each case, as shown in Table 1, the Venetian blind opened at a particular angle will be positioned 0.5 m away from inner glazing, Middle, and 0.5 m away from outer glazing, respectively.Table 1 shows the key to the various cases presented in Figs. 5(A–C) to 8(A–C). In each Figure, there are four images labelled, (a), (b), (c), and (d), respectively. The images labelled (a), (b), (c), and (d) provides a snapshot of the temperature distribution in the building at simulation time 25 s, 50 s, 75 s and 100 s, respectively. This is done for all the simulation cases (Case 2 to Case 13) representing the various Venetian blind positions and blind opening angles as presented in Table 1.

### 3.4 Model Validation - Description of the Surface Temperature Data

In the Mendeley Data repository [2], the data for the model validation results are also presented. These data are glazing surface temperatures in °C obtained from two thermocouples (TC) installed on the inner glazing surfaces. TC 59 was installed at a height of 1.6 m representing the thermocouple on the upper floor of the validation building model and TC 63 at a height of 4.8 m representing the thermocouple on the lower floor of the validation building model. In the comma-separated values file, there are three columns. The first column is the simulation time column in seconds and the other two columns captioned *‘Upper Floor Temperature°C THCP 59’,* and *‘Lower Floor Temperature°C THCP 63’*, respectively contain the respective glazing surface temperature data. The simulation time for each scenario was 1000 s with an interval of 1 s. The glazing surface temperature data is presented on the .csv file captioned Validation-Output_devc in the Mendeley Data repository [2].

### 3.5 Model Validation - Description of the Input and Output Source Code Files

In the Mendeley Data repository [2] is a folder captioned “Validation-Input”. This folder contains the FDS input file with the file extension “. fds” and provides the validation study input source code. This file can be opened using Microsoft Notepad editor.

## 4 Experimental Design, Materials, and Methods

### 4.1 Simulation Software and Approach

The model for the 5-storey building with multi-storey type DSF incorporating Venetian blinds was created in PyroSim Version 2021.1.0224. Uniform meshes (MESH01 and MESH02) for the building model were created in Fire Dynamics Simulator (FDS) version 6.7.5 for the simulation. In FDS, conservation equations for mass, momentum, and energy along with a combustion model based on mixture-fraction infinite fast reaction of lumped species was used to analyse the fluid dynamics, heat transfer, and combustion of Polyurethane GM27, the fuel selected for the fire. Smokeview (SMV) was then used to visualize the output of the simulation carried out using FDS.

### 4.2 Building Model Creation

The ground floor with dimensions 6 m × 6 m × 0.2 m thickness was created in PyroSim [3], using the Slab Obstruction tool. The component for the ground floor was then copied and pasted five more times to create the other floors of the building. The pieces were labeled First Floor, Second Floor, Third Floor, Fourth Floor Fifth Floor and Roof, respectively. Walls with 15.2 m height and 0.2 m thickness, were created using the Draw a Wall Obstruction tool in PyroSim [3].

The inner glazing of the DSF, 16 m high (including a 1 m high solar chimney) and 0.2 m thick was created using the Draw a Wall Obstruction Tool in PyroSim [3].The inner glazing was copied and shifted 1.4 m directly parallel to create the outer glazing of the DSF as shown in Fig. 5. This created a 1.2 m cavity between the inner and outer glazing of the DSF. An obstruction with 0.2 m thickness was created to stop air supply in -z and -y direction to make sure the air is only supplied through the proposed hole.

The ‘Draw a Wall Hole’ tool was used to create an opening of height 1.2 m to permit supply air into the DSF. A new group was created and named Venetian Blinds. *Via* the Model menu, a **New Obstruction** was created with **ID**, Blind and **Group**, Venetian Blinds. The values for the geometry of a Venetian Blind were then set in the dialog box to create the first blind. This component was then copied 70 times to create the group of Venetian blinds as shown in Fig. 6.

### 4.3 The Burner Fire

The **Burner** type was created by editing surfaces in the model menu. The Heat Release Rate Per Unit Area (HRRPUA) was set at 5000 kW/m^2^. The surface temperature of the fire along with its thermal boundary conditions were set in **Edit Surfaces**. The gas-phase reactions were set by editing the reactions in the PyroSim Libraries to add the Polyurethane GM27 fire. The geometry for the fire burner was created through **New Obstruction** in the **Model** menu. The top surface of the fire burner was set as the place where the fire appears. A **New Hole** was created in the model to simulate the broken window. In the geometry dialog box, the dimensions of the hole were set. Another **New Hole** to supply sufficient air for the burner fire was also created.

### 4.4 Mesh Creation

MESH01 was created for the fire room in the building model using **Edit Mesh**. Similar approach was used to create MESH02 for the DSF. The mesh boundaries were created, and redundant ones deleted. Fig. 7a and b shows 3D and 2D perspectives of the meshes created.

### 4.5 Surface Temperature Measurement

**New Thermocouple** was created to obtain glazing surface temperatures. Twenty-three (23) thermocouples were set up on the inner and outer glazing on each floor except the ground floor as shown in Fig. 8.

### 4.6 The Governing Equations

Conservation Eqs. (1) to (3) were applied in FDS to solve mass, momentum, and energy [4].

(1) $$Mass\;\to \;\frac{\;\partial \rho }{\partial t}+\nabla \cdot \left(\rho V\right)=0$$

(2) $$Momentum\;\to \frac{\;\partial }{\partial t}\left(\rho V\right)+\nabla \cdot \left(\rho \mathrm{VV}\right)=\rho f+\nabla \cdot \Pi_{\mathrm{ij}}$$

(3) $$Energy\;\to \;\frac{\partial E_{t}}{\partial t}+\nabla \cdot E_{t}V=\frac{\partial Q}{\partial t}-\nabla \cdot q+\rho f\cdot V+\nabla \cdot \left(\Pi_{\mathrm{ij}}\cdot V\right)$$

A constant $C_{v}$ = 0.1 was applied to the default Deardorff Model [5,6]. The combustion was based on the default Mixture Fraction Combustion Model in FDS applicable to Very-Large Eddy Simulation (V-LES) calculations. For an infinitely fast reaction, the HRR per unit volume presented in Eq. (4) was used [5].

(4) $${\dot{q}}^{{}^{\prime \prime \prime }}=-\sum_{a}{\dot{m}}_{a}^{{}^{\prime \prime \prime }}\Delta h_{f,a}$$

## 5 Model Validation - Modelling and Simulation Method

### 5.1 Building Model Creation

The ground floor with dimensions 4 m × 9 m × 0.2 m thickness was created in PyroSim [3], using the Slab Obstruction tool. The component for the ground floor was then copied and pasted to create the 2nd floors of the building. The pieces were labeled Lower floor and Upper floor, respectively. Walls with 6.6 m height and 0.2 m thickness, were created using the Draw a Wall Obstruction tool in PyroSim [3]. The inner glazing of the DSF, 6.6 m high and 0.2 m thick was created using the Draw a Wall Obstruction Tool in PyroSim [3].The inner glazing was copied and shifted 0.86 m directly parallel to create the outer glazing of the DSF as shown in Fig. 9. This created a 0.86 m cavity between the inner and outer glazing of the DSF. An obstruction with 0.2m thickness was created to stop air supply in -z and -y direction to ensure the air is only supplied through the proposed hole. The ‘Draw a Wall Hole’ tool was used to create an opening of height 1.2 m to permit supply air into the DSF. The building model was designed in according to work by Peng et al. [7] which the study was validated against. The simulation was run for 1000 s with an interval of 1 s.

### 5.2 The Burner Fire

The **Burner** type was created by editing surfaces in the model menu. The Heat Release Rate Per Unit Area (HRRPUA) was set at 2000 kW/m^2^ consistent with Peng et al. [7] The surface temperature of the fire along with its thermal boundary conditions were set in **Edit Surfaces**. The gas-phase reactions were set by editing the reactions in the PyroSim Libraries to add the Polyurethane GM27 fire. The geometry for the fire burner was created through **New Obstruction** in the **Model** menu. The top surface of the fire burner was set as the place where the fire appears. A **New Hole** was created in the model to simulate the broken window. In the geometry dialog box, the dimensions of the hole were set. Another **New Hole** to supply sufficient air for the burner fire was also created.

### 5.3 Mesh Creation

MESH01 was created for the fire room in the building model using **Edit Mesh**. Similar approach was used to create MESH02 for the DSF. The mesh boundaries were created, and redundant ones deleted. Fig. 10 shows 2D perspective of the meshes created.

## Ethics Statements

Not Applicable.

## CRediT authorship contribution statement

**Youxian Huang:** Investigation, Methodology, Writing – original draft, Validation. **Siegfried Yeboah:** Conceptualization, Supervision, Visualization, Writing – original draft, Writing – review & editing. **Jingjing Shao:** Conceptualization, Writing – review & editing, Funding acquisition.

## Declaration of Competing Interest

The authors declare that they have no known competing financial interests or personal relationships that could have appeared to influence the work reported in this paper.

## Data Availability

Simulation Dataset on Fire in the Cavity of Naturally Ventilated Double Skin Façade with Venetian Blinds (Original data) (Mendeley Data).
